# The Impact of Helminths on Colorectal Cancer: From Infections to the Isolation of Biotherapeutics

**DOI:** 10.3390/pathogens14090949

**Published:** 2025-09-20

**Authors:** Cuauhtémoc Ángel Sánchez-Barrera, Karen V. Fernandez-Muñoz, Mónica G. Mendoza-Rodríguez, María T. Ortiz-Melo, Jazmín A. Carrillo-Pérez, Miriam Rodríguez-Sosa, Luis I. Terrazas

**Affiliations:** 1 Unidad de Investigación en Biomedicina, Facultad de Estudios Superiores Iztacala, Universidad Nacional Autónoma de México, Avenida de los Barrios 1, Los Reyes Iztacala, Tlalnepantla 54090, Mexico; angel.casb94@gmail.com (C.Á.S.-B.); karen.fernandez@cinvestav.mx (K.V.F.-M.); teresaortiz@iztacala.unam.mx (M.T.O.-M.); jazmincarrillo71@gmail.com (J.A.C.-P.);; 2 Departamento de Biomedicina Molecular, Centro de Investigación y de Estudios Avanzados, Instituto Politécnico Nacional, Avenida Instituto Politécnico Nacional 2508, Ciudad de México 07360, Mexico; 3 Laboratorio Nacional en Salud, Facultad de Estudios Superiores Iztacala, Universidad Nacional Autónoma de México, Avenida de los Barrios 1, Los Reyes Iztacala, Tlalnepantla 54090, Mexico

**Keywords:** colorectal cancer, helminths, helminth-derived products, inflammation, biotherapeutics

## Abstract

Worldwide, colorectal cancer (CRC) is the third-most common cancer and the second-leading cause of cancer-related deaths. The inflammatory response initiated by pathogens, environmental and dietary factors, and inflammatory bowel diseases can promote the formation of colorectal tumors. The hygiene hypothesis proposes an inverse link between inflammatory diseases and early childhood exposure to pathogens, with a significant negative correlation between chronic inflammatory diseases and helminth infections. On the other hand, it is also known that several pathogens may influence or even cause the development of cancer, including helminth infections. How do helminth infections influence CRC outcomes? The existing literature presents two different perspectives. Experimental studies in CRC models suggest that helminths may accelerate disease progression and lead to worse outcomes (such as *Schistosoma* and *Trichuris* sp.), while others indicate that helminths could help reduce tumor burden (such as *Taenia* sp.). This review focuses on helminths’ pro- and anti-tumorigenic effects and their derivatives, specifically in CRC. We provide a comprehensive understanding of how helminths impact the macroscopic, histopathological, immunological, and molecular aspects of CRC.

## 1. Introduction

Colorectal cancer (CRC) ranks as the third-most common cancer globally and stands as the second-leading cause of cancer-related deaths worldwide. The incidence of CRC is rising among individuals younger than 50, particularly in high-income countries. This increase is associated with some risk factors, such as obesity, sedentary habits, and the consumption of antibiotics, red meats, alcohol, and tobacco. Another important factor is gut inflammation, such as inflammatory bowel diseases (IBD), including Crohn’s disease and ulcerative colitis (UC), that represent up to 18% increased risk of developing CRC [1,2].

The etiological origin of CRC can be categorized into three main types: hereditary cases, which account for 10% of all occurrences; familial history-related cases, comprising 25%; and sporadic cases, which represent 65% [3]. Currently, five genetic risk factors have been identified for hereditary CRC cases. These include early-onset CRC, a family history of cancer, deficient mismatch repair, multiple primary CRC tumors, and primary hereditary cancer syndrome associated with extra-colonic cancer. Each of these factors predisposes individuals to specific germline mutations, which can affect the clinicopathological phenotype, the family cancer spectrum, cancer penetrance, and long-term prognosis [4].

Colorectal tumors are classified in four consensus molecular subtypes (CMSs): (1) CMS1: hypermutated tumors characterized by microsatellite instability and strong immune activation, (2) CMS2: tumors exhibiting high levels of somatic copy number mutations and activation of the wingless-related integration site (WNT) and myelocytomatosis (MYC) signaling pathways, (3) CMS3: tumors demonstrating metabolic deregulation, and (4) CMS4: tumors associated with a mesenchymal phenotype, stromal infiltration, angiogenesis, activation of the transforming growth factor beta (TGF-β) signaling pathway, and a strong inflammatory response [5].

Chronic inflammation is a hallmark of cancer that drives tumorigenesis and tumor progression [6]. The inflammatory response initiated by pathogens, environmental and dietary factors, and IBD can promote the formation of colorectal tumors [7,8,9]. The accumulation of reactive oxygen species (ROS) released by immune infiltrating cells, particularly neutrophils, leads to DNA mutations in intestinal epithelial cells (IECs) [10]. Furthermore, the disruption of the intestinal barrier and the invasion of microbiota activate inflammatory factors associated with tumor progression, including the nuclear factor kappa-light-chain-enhancer of activated B cells (NFκB), interleukin (IL) 6/signal transducer and activator of transcription 3 (STAT3), cyclooxygenase 2 (COX2)/prostaglandin E2, and IL-23/T helper (Th) 17 signaling pathways [11].

The hygiene hypothesis proposes that a lack of exposure to microorganisms, mainly pathogens, during childhood may lead to an imbalanced immune response and increase the risk of developing inflammatory diseases such as allergies, asthma, and autoimmune diseases [12]. In recent years, certain helminth species have been linked to the hygiene hypothesis, indicating a negative correlation between the prevalence of chronic inflammatory diseases and some helminth infections. However, it is important to note that other helminths can exacerbate and even trigger inflammatory conditions [13]. In addition, it is also known that several pathogens may influence or even cause the development of cancer, such as human papillomavirus, which is linked to cervical cancer; *Helicobacter pylori*, associated with gastric cancer; and Hepatitis B and C viruses, which are related to liver cancer; protozoa, and less common helminth parasites have also been associated with the development of some neoplasia [14].

Helminths are a group of parasites that include nematodes (roundworms), trematodes (flukes), and cestodes (tapeworms) [15]. These organisms establish chronic infections and modulate the host’s immune system through their derived products [16]. Despite their diversity in sizes or life cycles, most immune responses triggered by helminth infections typically involve type 2 immunity, characterized by the production of cytokines such as IL-4, IL-5, IL-9, and IL-13. This response also recruits several innate and adaptive immune cells, including eosinophils, basophils, mast cells, dendritic cells (DCs), myeloid-derived suppressor cells, T regulatory cells (Tregs), type 2 innate lymphocytes (ILC2), alternatively activated macrophages (AAMs), and Th2 cells [17].

Although helminthiasis is recognized as a public health problem, many researchers employ various models to understand how helminths evade and modulate the immune response, and to develop therapies for inflammatory diseases. Experimental studies have consistently demonstrated a close association between helminth infections and favorable changes in the progression of autoimmune and inflammatory diseases, including inflammation-associated cancers such as CRC [18].

**Figure 1:**
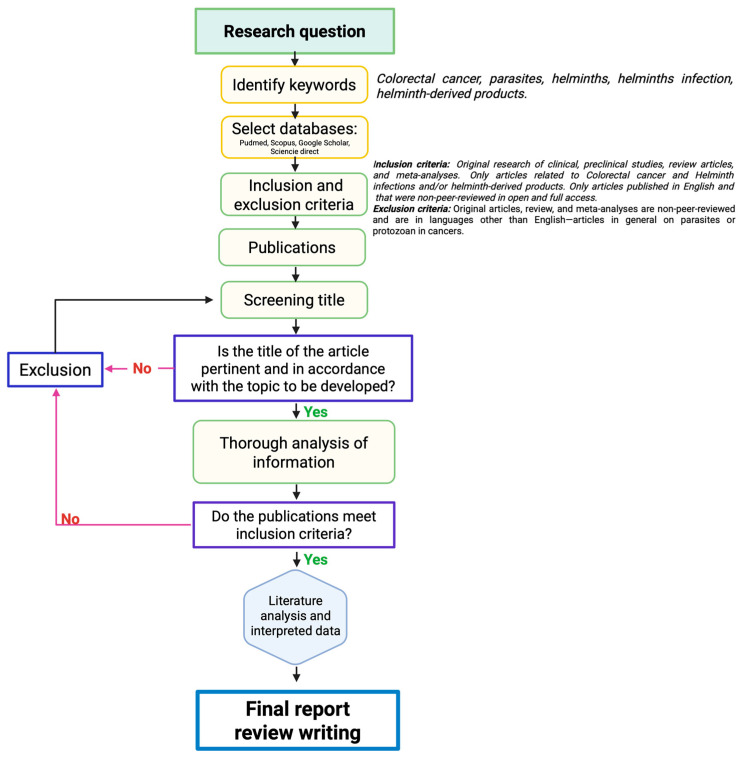
Methodological approach of the selection/exclusion of the reviewed literature. Black arrows indicate the consecutive steps for selecting publication, until the final report and review writing. The arrows in pink indicate the criteria that lead to the exclusion of works non included in the final report. Purple frames indicates critical steps for deciding to selection or exclusion works.

How do helminth infections influence cancer outcomes? The existing literature presents two different perspectives. Experimental studies in CRC models show that helminths may accelerate disease progression and lead to worse outcomes, while others indicate that helminths could help reduce tumor burden [19]. The mechanisms behind these effects are currently under intensive research and vary according to the type of cancer and the type of helminth involved in the study. To avoid confusing generalizations, this review focuses on the pro-tumorigenic and anti-tumorigenic effects of helminths and their derivatives, specifically in CRC (Table 1). We aim to provide a comprehensive understanding of how helminths affect the macroscopic, histopathological, immunological, and molecular aspects of CRC by reviewing the existing literature. The keywords “colorectal cancer”, “parasites”, “helminths”, “helminth infection”, and “helminth-derived products” were searched in the selected databases: PubMed, Scopus, Google Scholar, and Science Direct. The inclusion criteria specifically targeted studies that explored the relationship between CRC and helminths, while excluding works that examined other types of cancers and those that did not involve helminthic parasites, such as protozoa (Figure 1).

## 2. The Impact of Helminth Infections on Colorectal Cancer

Helminthiases are among the most prevalent infections worldwide, affecting nearly 24% of the global population [42]. The widespread presence of helminth infections allows them to coexist with other chronic diseases, including cancer. A recent systematic review analyzed studies conducted in 26 countries and found that approximately 20.79% of patients with CRC were infected with helminthic parasites, with Schistosoma species being the most common culprits [43]. This suggests that a significant number of CRC patients may be susceptible to developing helminth infections. Therefore, it is essential to understand the impact of helminths on the development of CRC to prevent worse outcomes and to explore any potential beneficial effects they may have.

There are several general mechanisms by which helminth infections and their products may impact CRC development either positively or negatively: (1) indirect bypass affecting the host’s immune responses, (2) direct carcinogenic or anti-carcinogenic effect, (3) acute or chronic histo-mechanical damage, (4) altering the homeostasis of the microbiome, and (5) antigenic cross reactivity given a molecular mimicry.

**Figure 2:**
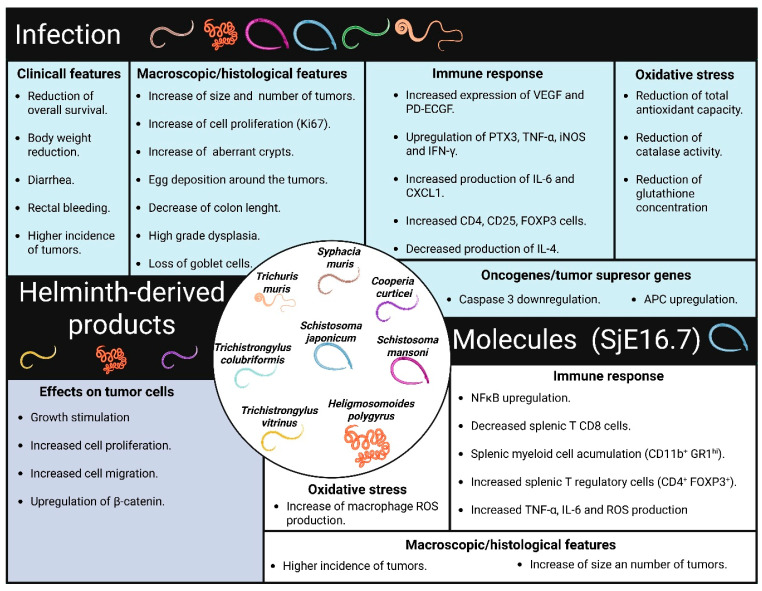
Physiological changes associated with the protumor effects of helminths. Specific helminth infections contribute to tissue damage and adverse clinical outcomes in CRC. These infections trigger physiological changes in their hosts, including alterations in immune response and antioxidant mechanisms. The protein SjE16.7, which is derived from *S. japonicum*, has similar effects on the development of CRC. Additionally, helminth-derived products can directly affect tumor cells, promoting tumor growth.

**Figure 3:**
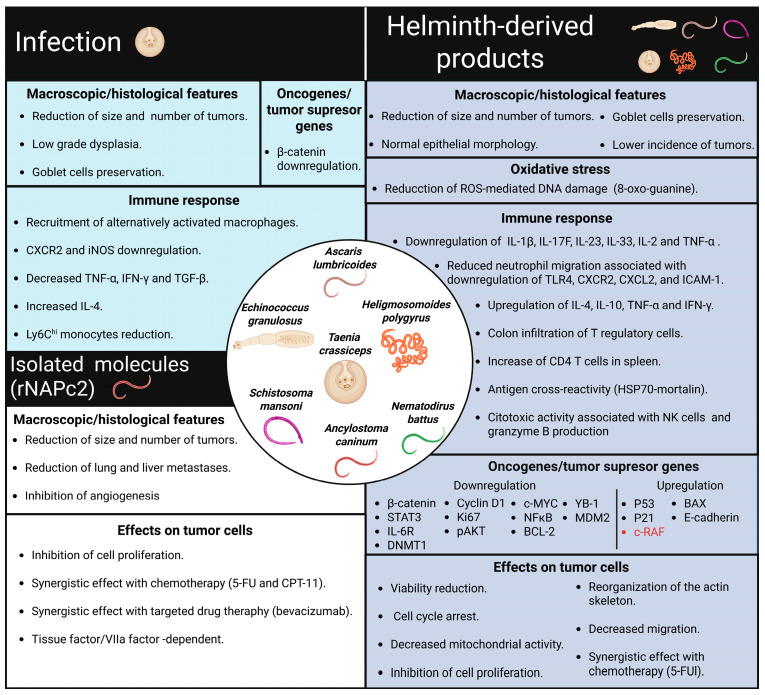
Physiological changes are associated with the antitumor effects of helminths. Infection with *T. crassiceps* improves CRC outcomes by inducing an anti-inflammatory immune response in the host. Similarly, several helminth-derived products have been shown to reduce the incidence of colon tumors. Additionally, these products exhibit synergistic effects when combined with chemotherapy, directly influencing tumor cells by regulating oncogenes and tumor suppressor genes. Furthermore, the rNAPc2 recombinant protein, obtained from *Ancylostoma caninum*, has been found to reduce tumor growth and metastasis in different mouse models.

The first section examines the negative and positive effects of helminth infections on the development of CRC. The second section, entirely experimental, discusses the impact of helminth-derived products at different stages of CRC onset in preclinical assays or in in vitro models, focusing on the mode of action involved in this disease. Notably, the primary mechanisms discussed involve modulating the host’s immune response, likely due to the characteristics of the in vivo models used in the reviewed literature (Table 1, Figure 2 and Figure 3).

### 2.1. Pro-Tumorigenic Effects of Helminthic Infections in CRC

Few helminth parasites have been identified as direct causes of cancer development. In its monographs on identifying carcinogenic hazards to humans, the International Agency for Research on Cancer (IARC) classifies infections with the helminths *Opisthorchis viverrini* and *Schistosoma haematobium* as Group 1 agents, which are proven to promote bile duct cancer. Additionally, infection with *Schistosoma japonicum* is classified as a group 2B agent, indicating that it is possibly carcinogenic to humans, mainly causing liver cancer, and has been associated with the development of CRC [14]. In this section, we discuss several findings suggesting that the potential unfavorable effect of helminth infections on cancer development may be more related to their immunological or bypass effects (Figure 2).

Patients with CRC who also show a *Schistosoma* sp. infection exhibit reduced overall survival rates [25,43]. This outcome may be related to egg deposition around the tumors, as reported in different cases of *S. japonicum* infection [21,26]. This phenomenon leads to an upregulation of vascular endothelial growth factor (VEGF) and platelet-derived endothelial growth factor (PD-ECGF) expression [22]. Notably, the increased expression of VEGF has been associated with the pro-angiogenic effects that promote tumor progression, migration, and metastasis [23].

Chronic inflammation is a critical factor that contributes to the development of CRC [11]. In this context, helminth infections can trigger inflammatory responses that may promote tumor growth in CRC. For example, infection with *Schistosoma mansoni* in mice has been shown to cause several pathological changes, including distortion of colonic crypts, glandular and mucosal dysplasia, nuclear hyperchromasia, and serration of the surface epithelium [44]. *S. mansoni*-infected mice also exhibited elevated serum levels of pentraxin 3 (PTX3) and tumor necrosis factor-alpha (TNF-α), concurrently with local upregulation of inducible nitric oxide synthase (iNOS) and TNF-α in the colon [44]. While there is no direct evidence that these molecules are responsible for the pathological changes associated with *S. mansoni* infection, studies have indicated that elevated levels of PTX3 are linked to decreased overall survival in post-surgery CRC patients [24]. Additionally, high levels of TNF-α correlated with more advanced tumor stages in CRC patients [45], while the overexpression of iNOS is known to promote tumor angiogenesis and metastasis [46].

In intestinal living helminths, other mechanisms may influence CRC outcomes. In a study using the alkylating agent azoxymethane (AOM) and the polysaccharide dextran sulfate sodium (DSS) model, mice infected with *Heligmosomoides polygyrus,* one day after AOM treatment, exhibited accelerated weight loss and a higher incidence of colonic tumors. In contrast, mice infected at week 8 after the initiation of AOM/DSS induction did not exhibit significant differences in weight, and tumor incidence [47]. These findings suggest that *H. polygyrus* infection may promote colorectal tumorigenesis only at early stages of the disease, when this parasite induces mechanical harm upon attachment to the intestinal wall (see below).

Accordingly, *H. polygyrus* worsened the acute UC model induced by DSS, causing significant weight loss and a higher disease activity index compared with their uninfected counterparts. Moreover, histological analysis revealed more substantial changes in the colon, including shortening, loss of structural integrity, depletion of goblet cells, and increased recruitment of inflammatory cells [47]. Molecularly, the mechanism increasing UC by *H. polygyrus* involves the downregulation of IL-4 production and increased levels of IL-6 and chemokine (C-X-C motif) ligand 1 (CXCL1). Interestingly, blocking IL-6 and CXCL1 reduced the severity of UC in infected mice. In contrast, the small intestine of infected mice treated with DSS did not show any pathological changes. Additionally, lower levels of IL-6 and CXCL1 were observed in the ileums of infected mice with DSS-induced colitis compared to their uninfected counterparts [47]. These findings are consistent with studies demonstrating a critical role of IL-6 in the AOM/DSS model, where *IL-6* knock-out mice showed reduced tumor formation [20]. Additionally, CXCL1 expression correlated with decreased overall survival in CRC patients [48]. Notably, *H. polygyrus* larvae encyst, molt, and emerge as adult worms in the small intestine, indicating that this worm triggers a local immunosuppressive response. These findings suggest that helminth niches play a crucial role in shaping the effects of helminthiasis on CRC outcomes (discussed below).

Another case that highlights how intestinal helminths can affect CRC outcomes is the infection with *Trichuris muris*. This parasite establishes itself in the large intestine and caecum of the host, where it induces inflammatory conditions characterized by the production of cytokines, such as IL-6, TNF-α, and interferon gamma (IFN-γ), which generate, after chronic infection, pathological changes like those observed in the AOM model of CRC. Thus, on day 80 post-infection, *T. muris* was found to promote colon hyperplasia, the development of aberrant crypts, pre-adenomas, and an influx of inflammatory cells into the colonic lamina propria [49]. When these chronically infected animals were exposed to AOM, they developed increased levels of neoplasia scores within the colon. These results suggest the need to investigate the immune response in the colon during infection with *T. muris* to elucidate a potential mechanism that favors CRC.

Approximately 25% of CRC patients have a family history of the disease. Familial adenomatous polyposis syndrome (FAP) is characterized by germline mutations in the adenomatous polyposis coli (*APC*) gene, which result in an almost 100% risk of developing CRC [28]. Interestingly, there are studies on how helminth infections impact this condition. Thus, studies involving the *APC*^min/+^ mouse model, the loss of *APC* heterozygosity induced by environmental factors, such as pathogen infections, can accelerate tumor formation in the gastrointestinal tract [50]. *APC*^min/+^ mice infected with *T. muris* for 18 and 42 days exhibited tumor formation in the large intestine. This tumor development was prevented when CD4^+^, CD25^+^, FOXP3^+^ Tregs were depleted, indicating that *T. muris* infection influences CRC through immunosuppression mediated by Tregs [49]. These findings suggest that in patients with a family history of FAP, *T. muris* infection may trigger or enhance colonic tumor growth through Treg-mediated immunosuppression.

As previously mentioned, the niches where helminths reside may influence the effects of helminthiasis on CRC outcomes. To explore this further, an experiment was conducted using the *APC*^min/+^ mouse model to compare the effects of two different types of helminths, the small intestine-dwelling *H. polygyrus* and the colon-dwelling *T. muris,* on CRC outcomes. Unlike *T. muris* infection, *H. polygyrus* infection did not promote tumor formation in the small intestine [49]. Interestingly, the results showed that these parasites did not promote tumor formation in their regular habitats. Still, they did favor tumor development in gastrointestinal areas that were distant from their usual niches. These findings align with previous studies indicating that *H. polygyrus* does not lead to tumor formation in the small intestine, its primary habitat, but does promote tumor formation in the colon. Additionally, lower levels of IL-6 and CXCL1 were observed in the ileum of *H. polygyrus*-infected mice treated with DSS, compared with the levels found in the colon of only infected mice [47]. These findings suggest that intestinal living helminths can trigger opposing immune responses in both their primary habitats and in distant areas. This opposing activity may either promote or inhibit CRC tumor progression, depending on the characteristics of the specific microenvironment.

Another exclusive intestinal helminth infection is that caused by *Syphacia muris.* A previous infection with this nematode resulted in downregulation of caspase 3 and APC, and the upregulation of COX2, promoting epithelial cell proliferation and increasing aberrant crypt formation in a CRC model induced by 1,2-dimethylhydrazine (DMH). Antioxidant assays revealed a reduced total antioxidant capacity in the host, which was associated with lower catalase activity and glutathione levels. These changes were linked to colonic tissue damage and an increased number of colonic tumors [51]. Oxidative stress and inflammation are closely interconnected and can exacerbate each other. During chronic inflammation, the production of ROS increases due to the recruitment of phagocytes, such as neutrophils [27]. These ROS can cause DNA damage through oxidation, leading to the accumulation of driver mutations. The most common ROS-induced mutation is the G-C transversion, which occurs due to the oxidation of guanine, resulting in the formation of 8-oxo guanine [52]. In response to oxidative stress, several antioxidant mechanisms are activated. This includes the upregulation of antioxidant enzymes, such as superoxide dismutase, catalase, thioredoxins, and glutathione peroxidase [27], which appear to be downregulated by *S. muris* infection [51]. Thus, these findings suggest that certain intestinal helminth infections may disrupt the balance between oxidative stress and antioxidants, ultimately leading to colon tissue damage and favoring the development of CRC.

At least four different helminth infections, caused by *Schistosoma* species, *T. muris*, *S. muris*, and *H. polygyrus*, have been linked to the initiation or exacerbation of colorectal tumors (Figure 2). Additionally, several physiological changes have been observed that are associated with the tumor-promoting effects of these helminth infections. These changes include the upregulation of angiogenic factors, disruption of antioxidant mechanisms, and the promotion of chronic inflammation (Figure 2). However, further research is needed to clarify the mechanisms by which helminth infections contribute to gastrointestinal tract dysfunction and the development of CRC.

### 2.2. Anti-Tumorigenic Effects of Helminthic Infections

The gut microbiota is an ecosystem made up of bacteria, fungi, viruses, Archaea, and parasites that interact with IECs to maintain homeostasis. This ecosystem plays a crucial role in the fermentation of non-digestible carbohydrates, nutrient absorption, vitamin synthesis, and modulation of host immunity. When the function or composition of the microbiota is disrupted, this condition is referred to as dysbiosis, which is associated with several diseases, including CRC [53].

In CRC patients, dysbiosis is characterized by an increase in pathogenic bacteria, including *Fusobacterium nucleatum*, *Veillonella*, *Prevotella*, and *Streptococcus*. These bacteria promote tumor initiation and progression through several mechanisms, such as genotoxin-mediated mutagenesis, modulation of oncogenic cell signaling pathways, alteration of chemotherapeutic metabolism, and induction of inflammation [54].

It is not hard to think that gastrointestinal-living helminths may modulate the microbiome in such tissue. Several reports indicate specific alterations in the microbiota in humans and murine models. Different species of all helminth classes have been associated with changes in the gut microbiome [55], but the impact of such alterations on an individual’s health is incompletely understood. Mechanistically, helminths may modify the microbiome by altering the metabolism of their hosts, impacting epithelial junctions, or even directly eliminating bacterial populations susceptible to the antimicrobial activities of their ES products through the induction of antimicrobial peptides and mucus production from the hosts [56]. Currently, there is no direct evidence linking changes in the microbiota caused by helminths to the development of CRC. However, it has been suggested that the eradication of helminths from human populations may be associated with an increase in dysbiosis and IBD [53]. Additionally, some studies indicate that certain helminth infections may protect their hosts from intestinal inflammation by altering the microbiome. Studies in mice infected with *T. muris* or *H. polygyrus* led to a reduction in the pathogenic bacterium *Bacteroides vulgatus* and an increase in the population of beneficial *Clostridium species*, thereby preventing intestinal inflammation [57]. Furthermore, male *S. mansoni* worm infections were found to protect mice from chemically induced colitis. In contrast, infection with both male and female worms exacerbated symptoms. Differences were observed in the microbiota of mice infected with male worms compared to those infected with both sexes, where male *S. mansoni* infection inhibited the growth of colitogenic bacteria. Notably, the protective effects of male *S. mansoni* worms were lost with the administration of antibiotics, suggesting a central role of bacterial microbiota [58]. Interestingly, these microbiome alterations do not affect the expulsion of the helminth *Hymenolepis diminuta* from the intestine [59]. Similarly to *S. mansoni*, eliminating gut microbiota by using antibiotics or in germ-free mice inhibits the protective effect of *H. diminuta* on ulcerative colitis [60], indicating that helminth-induced alterations in the microbiota are critically involved in the helminth control of intestinal inflammatory diseases. However, whether helminth-derived products induce such microbiome modulation is unknown, nor whether extraintestinal helminth infections may impact the colon microbiome and therefore CRC outcomes. Overall, these findings indicate that certain helminths may promote the growth of beneficial bacteria, which help reduce chronic inflammation in the intestine, a well-known risk factor for the development of CRC. However, further research is needed to understand better the relationship between helminths, microbiota, and CRC.

*Taenia crassiceps* is a cestode parasite that, in its adult stage, lives in the intestine of canids. Its larval stage (metacestode) can live in the muscle, brain, and peritoneal cavity of rodents. Intraperitoneal infection with the larval stage of *T. crassiceps* is characterized by the induction of a dual immune response. In the early acute stages, *T. crassiceps* infection triggers a temporary Th1-type immune response marked by high levels of IL-2, TNF-α, IFN-γ, nitric oxide, and IgG2a antibodies. However, in the chronic stages of infection (6 weeks or more), this Th1 response is replaced by a strong Th2-type immune response, which features elevated levels of IL-4, IL-5, IL-10, IL-13, the presence of AAMs, and high levels of IgG1 and IgE [61].

In the AOM/DSS CRC model, mice pre-infected six weeks earlier with *T. crassiceps* developed a lower incidence of colon tumors and reduced severity of CRC. Remarkably, 20% of the infected mice were tumor-free, exhibiting low-grade dysplasia and normal goblet cell counts. The remaining 80% of the infected mice had significantly fewer and smaller tumors than non-infected mice. Furthermore, the mice infected with *T. crassiceps* showed reduced levels of β-catenin in the colon [62], a marker for the advancement of CRC. Indeed, approximately 60–80% of CRC patients experience abnormal activation of the WNT/β-catenin signaling pathway. Elevated levels of nuclear β-catenin expression in CRC patients are associated with decreased overall survival rates, as the activation of β-catenin triggers the transcription of target genes that promote tumor cell proliferation [32]. Interestingly, similar results were reported in other solid tumor models, where mice infected with *Trichinella spiralis* for 35 days displayed a reduced size of liver tumors [63].

Unlike protumor helminth infections that promote tumor growth and trigger an inflammatory response in the intestine, mice pre-infected with *T. crassiceps* showed a reduced inflammatory infiltrate in the colonic lamina propria. This was associated with the low levels of the chemokines C-X-C chemokine receptor 2 (CXCR2) and C-C chemokine receptor 2 (CCR2), resulting in decreased recruitment of neutrophils and Ly6C^hi^ monocytes. In contrast, anti-inflammatory cell populations, such as AAMs, were detected in the colon tissue. Additionally, levels of inflammatory cytokines, including TNF-α, IFN-γ, and the expression of iNOS, were reduced, while the anti-inflammatory cytokine IL-4 increased significantly [62]. To further assess whether *T. crassiceps* infection decreases intestinal inflammation, infected mice were induced to develop acute colitis through DSS treatment. *T. crassiceps* infection effectively reduced clinical symptoms, including weight loss and disease activity index. Moreover, the infected mice maintained their colon size and preserved colonic epithelial structure. These beneficial effects were linked to the recruitment of AAMs to the colonic lamina propria. Furthermore, transferring AAMs isolated from *T. crassiceps*-infected mice improved symptoms of DSS-induced colitis in naïve receptor mice [64].

This section summarizes the connection between the immune response triggered by helminth infections and their significant impact on the development of CRC, particularly in the early stages of the disease. The reviewed literature suggests that particular helminth species promote a local inflammatory response or a localized injury that actively supports protumor activity, thereby facilitating the initiation of CRC (Figure 2). In stark contrast, other helminth infections, such as *T. crassiceps*, elicit strong global anti-inflammatory responses linked to antitumor effects that may unfueled the formation of colorectal tumors (Figure 3).

Based on these observations, we hypothesized that a delicate balance between inflammatory and anti-inflammatory responses in the host influences the effects of helminth infections on tumorigenesis in the early stages of sporadic CRC. In this context, helminth infections that stimulate an inflammatory response in the intestine may lead to tissue damage and the formation of tumors in the colon. Conversely, an anti-inflammatory response could help modulate intestinal inflammation, reducing tissue damage and preventing tumorigenesis in the colon.

The precise mechanisms by which helminth infections trigger inflammatory or anti-inflammatory responses are still being elucidated. Intestinal helminths attach themselves to the epithelium through specialized structures, such as the scolex, which results in tissue damage [15]. This tissue injury promotes the release of alarmins, such as IL-25, IL-33, and thymic stromal lymphopoietin (TSLP), from IECs. These alarmins activate ILC2, which activate the Th2 immune response and promote tissue repair. The impact of alarmins varies based on the parasite species, cellular source, and the immune microenvironment. Evidence suggests that alarmins are important for both host protection and tissue pathology [65]. Therefore, it is essential to understand how the stimulation of alarmins by helminths affects outcomes in CRC.

## 3. The Impact of Helminth-Derived Products on Colorectal Cancer

While certain helminth infections may have beneficial effects on CRC, using whole live parasites as helminth therapy is impractical and could pose a health risk to patients, as several studies have indicated. Although helminthiasis is generally not fatal, it can lead to several health issues, including anemia, malnutrition, stunted growth, internal organ blockage, and organ damage. Additionally, it can cause immune-related problems such as chronic inflammation, fibrosis, granuloma formation, and immunosuppression, depending on where the parasite is located [66]. Despite the risks associated with using whole helminths as therapy, six clinical trials have been conducted to date to evaluate the efficacy and safety of treating IBD with *Trichuris suis* eggs (TSO) (NCT01433471, NCT03565939, NCT01576471, NCT01434693, NCT01279577, NCT03079700). The current role of TSO as a treatment for IBD remains uncertain. Some clinical trials have indicated a reduction in disease severity among patients without serious adverse effects. However, meta-analysis studies suggest that no statistically significant results have been observed. Additionally, the small size of the study cohorts makes it challenging to draw definitive conclusions [67].

To avoid the potentially serious adverse effects of whole and long-term helminth therapy, recent studies have focused on analyzing helminth-derived products as an alternative. Helminths excrete and secrete several products, including metabolites, glycans, lipids, nucleic acids, fatty acids, glycolipids, proteins, and glycoproteins. Helminth-derived products primarily interact with the host immune system in two principal ways: either by inducing a protective immune response or by evading the host immune response. Currently, these effects are studied as potential immunomodulatory and biotherapeutic drugs for chronic inflammatory diseases, including inflammation-associated cancers [68]. In this section, we reviewed the effects of helminth-derived products in CRC progression in in vitro and pre-clinical assays.

### Anti-Tumorigenic Effects

In 1970, Berton Zbar and his colleagues demonstrated that immunization with the attenuated Bacillus Calmette–Guerin (BCG) could stimulate the immune cells to target cancer cells. Today, BCG is an approved treatment for bladder cancer [69]. In this context, recent research has utilized pathogen-derived products as biotherapeutics to treat various diseases. Here, we reviewed evidence showing that helminth-derived products can enhance the immune response against CRC cells and potentially alter the tumor microenvironment to inhibit cancer cell proliferation.

In a heterotopic syngeneic mouse model of CRC, researchers transplanted the CRC cell line CT26 (5 × 10^5^ cells) into the backs of BALB/c mice, and five days later, they administered three injections of hydatid cyst fluid (HCF) from *Echinococcus granulosus* or the 78 KDa fraction of HCF every two weeks. These treatments resulted in a significant reduction in the mean tumor area. Furthermore, while serum levels of IL-2 decreased, the levels of TNF-α and IFN-γ increased. Notably, high levels of IgG were observed one week after the last injection. In contrast, untreated mice showed a higher mean tumor area with low levels of TNF-α and IFN-γ [70].

Immunizing mice with HCF before tumor induction using the heterotopic syngenic model of CRC (CT26 colorectal cells) reduces tumor incidence by 40%. Moreover, administration of HCF at 4-, 7-, and 10-day post-tumor induction increases mouse survival by 40%. Additionally, anti-HCF antibodies generated in rabbits can target CT26 CRC cells. Mass spectrometry analysis revealed that these anti-HCF antibodies also recognize the heat shock protein 70 (HSP70), found in *E. granulosus,* which shares 60% homology with mortalin. This protein binds to P53 and inhibits its antiproliferative activity [34].

Given that antibodies against *E. granulosus* cross-react with mortalin, it is essential to conduct further experiments to determine whether these antibodies contribute to the antitumor effects triggered by HCF and how these antibodies may cross both cell and nuclear membranes to bind to their putative target.

Helminth-derived products are significant sources of parasite antigens recognized by the host immune system. The presentation of these antigens leads to the production of antibodies against helminth products [16]. Interestingly, cancer cells and helminths share similar mucin-type *O*-glycan compositions, including α-N-acetylgalactosamine-O-serine/threonine, sialyl-Tn, or Thomsen–Friedenreich antigens. Consequently, antibodies against helminth products can cross-react with cancer cells [35].

Helminths influence the host’s immune system through their derived products [16]. In this context, experiments conducted using the DMH CRC mouse model demonstrated that a double dose of autoclaved *T. spiralis* antigens (ATSA, 70 mg/kg) or autoclaved *S. mansoni* antigens (ASMA, 5 μg/kg) administered during the 12th week post-cancer induction, resulted in increased overall survival rates of 60% and 80% by the 20th week of the study, respectively [71]. Concomitantly, groups of mice treated with ASMA or ATSA displayed lower IL-17 levels in serum compared with cancer control mice. Moreover, these treatments also increased the serum concentration of IL-10, CD4 T cells in the spleen, and favored the recruitment of Tregs into the colon. While ASMA-treated mice showed a reduction in colon tumor size, the number of neoplastic lesions, and the average lesion size per mouse, the ATSA-treated group showed an increased average lesion size compared to the untreated control group [71]. Thus, ATSA and ASMA treatments increase serum IL-10 levels and recruit FOXP3+ Tregs to the colon, having opposite effects on CRC outcomes. This suggests that helminth-derived products not only affect CRC progression through immunomodulation but also involve other mechanisms, including direct effects on tumor cells (discussed below).

Other helminth-derived molecules have also been tested against CRC. The larval phase of *T. crassiceps* is known by the release of excreted/secreted (ES) factors that alter the response of innate immune cells of mice and humans [61]. These molecules are mainly glycoproteins >50 kDa. Using the AOM/DSS model of CRC, a relatively early (before the third DSS cycle, when colon tumors initiate their growth) 200 μg administration of *T. crassiceps* excreted/secreted products (TcES) three times a week, starting on day 26 after induction and continuing until day 65, resulted in the inhibition of colonic tumor formation in 45% of treated mice. In the remaining 55% of cases, the developed tumors were smaller than those in untreated mice. Furthermore, mice receiving TcES exhibited normal colon epithelium morphology and maintained normal goblet cell counts [38].

The underlying mechanism of this inhibition involves a reduction in STAT3 phosphorylation through the downregulation of the IL-6 receptor (IL-6R). The inhibition of the IL-6-STAT3 signaling pathway was confirmed by a reduction in the transcription of STAT3 target genes, including DNA methyltransferase 1 (DNMT1) and Cyclin D1 [38]. Cyclin D1 is a proto-oncogene that plays a crucial role in the transition from the G1 phase to the S phase during cell cycle progression, thereby favoring tumor cell proliferation [39]. DNMT1 is closely associated with the activation of β-catenin and its transcriptional activity [72]. Notably, the nuclear translocation of β-catenin was reduced in TcES-treated mice [38].

Like *T. crassiceps* infection, treating CRC with TcES also reduces intestinal inflammation. CRC mice treated with TcES exhibited decreased colon tissue levels of pro-oncogenic cytokines, including IL-1β, IL-17F, IL-23, and IL-33. Additionally, a reduction in CXCR2 and intercellular adhesion molecule 1 (ICAM-1) was associated with decreased tumor neutrophil infiltration [38]. In recent years, the protumor effects of neutrophils have been described, including the increased production of ROS [73,74]. Interestingly, lower levels of 8-oxo guanidine were found in TcES-treated mice compared to untreated ones [38]. Together, these data suggest that the reduction in neutrophil infiltration is associated with decreased ROS-mediated DNA damage. Another interesting and related finding was that TcES-treated mice also displayed a reduced activation of NFκB signaling. Consequently, the expression of target genes such as TNF-α and the anti-apoptotic protein B-cell lymphoma 2 (BCL-2) was downregulated [38]. Therefore, TcES downregulates inflammation-related signaling pathways involved in CRC development.

These findings demonstrate that the immunomodulatory properties of helminth-derived products can be harnessed as a prophylactic strategy to reduce intestinal inflammation and prevent tumor formation effectively. Furthermore, pursuing new treatment strategies, particularly combining chemotherapy with immunotherapy, is essential to improve patient outcomes [75]. In this context, mice with AOM/DSS-induced CRC at advanced stages (after the third DSS cycle) were treated with TcES (200 μg) in combination with 30 mg/kg of the chemotherapeutic agent 5-Fluorouracil (5-FU). Remarkably, mice receiving the combinatory treatment (TcES+5-FU) exhibited a 70% reduction in tumor load. In contrast, the individual treatments with 5-FU or TcES alone resulted in tumor reduction of only 15% and 40%, respectively [76].

The effective antitumor effect of combined treatment (TcES+5-FU) was associated with increased infiltration of cytotoxic cells, such as natural killer cells (NK), which promote tumor cell apoptosis by releasing granzyme B. In addition, this combined treatment also reduced the expression of murine double minute 2 (MDM2), a P53 repressor, leading to the upregulation of P53 and P21, suggesting that combined treatment leads to cell cycle arrest of tumor cells [76]. This effect may provide an opportunity for the recruited immune cells to exert their cytolytic function. Therefore, such evidence suggests that ES helminth products can be promising biotherapeutic adjuvants for chemotherapy.

The regulation of proto-oncogenes and tumor suppressor genes observed in the treatment of ES helminth products raises the question of whether there is direct interaction between helminth ES products and CRC tumor cells. Our next section will address this question.

## 4. The Impact of Helminth-Derived Products on Colorectal Cancer, Beyond the Immune System

The revised reports above have primarily focused on the impact of helminths on CRC through modulation of the immune system. However, since intestinal helminths reside in the gastrointestinal tract, they come into direct contact with epithelial and tumor cells. This raises the question: what effects does this interaction have? To understand this, it is necessary to consider the two main models that explain the cellular origin of CRC. The first is the bottom-up model, which is associated with hereditary or familial history factors and suggests that CRC originates from stem cells at the bottom of intestinal crypts. The second is the top-down model, linked to sporadic cases of CRC, which posits that the cancer arises from stem-like cells at the top of the crypts [40]. Recent studies indicate that inflammatory CRC may originate from well-differentiated cells, such as Paneth cells [77]. However, this hypothesis is still under investigation.

Several effects of helminths on the gut epithelium have been documented. These effects include increased epithelial permeability, degradation of mucus, and mechanical damage, all of which promote alarmin production. Additionally, it has been hypothesized that helminths influence stem cell differentiation towards specific lineages, which helps maintain epithelial integrity while enhancing the persistence of the worm. In response, host epithelial cells can activate various signaling pathways to defend against these infections. These protective mechanisms include mucus production, cellular proliferation, and activation of the immune response [78]. This section examines the potential impact of the interaction between helminth-derived products and tumor cells on outcomes in CRC.

### 4.1. Pro-Tumorigenic Effects of Helminth-Derived Products

Few studies have reported direct pro-tumorigenic effects of helminth-derived molecules on CRC tumor cells. For example, exposure of the HT29-D4 human CRC cell line to 1 μg/mL of conditioned medium from *Trichostrongylus colubriformis* increased cell proliferation. Treating this conditioned medium with heat, acid hydrolysis, or precipitation using trichloroacetic acid reduced the cell proliferation of HT29-D4. This suggests that *T. colubriformis* secreted proteins may enhance the proliferation of colorectal tumor cells [79]. Similarly, stimulating HT29-D4 cells with 0.1 μg/mL of conditioned medium of *Trichostrongylus vitrinus* and 1–5 μg/mL of conditioned medium from *Cooperia curticei* also led to increased cell proliferation [30].

Cell migration is a key step in cancer invasion and metastasis, reflecting the pathological grades of malignancy associated with cancer cells [6]. Studies using the mouse CRC cell line CT26 revealed that exposure to 10 μg of *H. polygyrus* antigens or 10 μg of *H. polygyrus* excreted/secreted products (HES) increased cell migration and β-catenin expression. Interestingly, the effects of these antigens on human CRC cells contrast with those observed in CT26 cells (discussed below) [29]. Although this evidence suggests that helminth-derived products may promote cell migration and proliferation in CRC cells, the underlying mechanisms remain unclear. This highlights the need for further research to understand the underlying mechanisms.

### 4.2. Anti-Tumorigenic Effects of Helminth-Derived Products

The direct antitumor effects of helminth-derived products on CRC cell lines have been studied in several helminth species and in greater depth. Initial reports indicate that ES products from *Nematodirus battus* inhibited the proliferation of HT29-D4 cells [30]. Also, the *Ascaris lumbricoides* ES products (ALES) inhibited HCT116 cell proliferation [37]. However, the direct antiproliferative mechanisms of *N. battus* and *A. lumbricoides* products remain unclear.

Further investigations have explored the mechanisms behind the direct antitumor effects of helminth-derived products in several CRC cell lines. Research using the mouse CT26 cell line indicated that cells treated with egg antigens from *S. mansoni* showed no significant effects. However, treatment with 10 μg of HES decreased viable cell counts, reduced DNA synthesis, and mitochondrial activity. Conversely, treatment with 10 μg of antigens from *H. polygyrus* reduced viable cell numbers but did not affect DNA synthesis or mitochondrial function. Both treatments increased the expression of the proteins P53 and P21, suggesting that these proteins might contribute to the antiproliferative effects of *H. polygyrus* antigens. As previously mentioned, these treatments also induced cell migration in the CT26 cell line [29].

On the other hand, experiments using the human cell line HCT116 demonstrated that HES reduced cell viability and DNA synthesis without impacting mitochondrial activity. The antigens from *H. polygyrus* did not affect the HCT116 cells. However, like the CT26 cells, both treatments induced the upregulation of P53 and P21 in HCT116 cells. Unlike CT26 cells, exposure to *H. polygyrus* antigens and HES decreased cell migration and β-catenin expression in HCT116 cells [29].

These observations highlight the differences in the anti-tumorigenic activity of antigens among helminth species and the variations between total and ES antigens. Such differences may result in varying impacts on CRC cells. It is important to consider that helminth-derived products might induce species-specific responses, exhibiting a range of effects on CRC cells depending on their origin.

Recent studies, like those conducted using *H. polygyrus*, have demonstrated the direct impact of HCF from *E. granulosus* on both mice and human CRC cells. Exposure of the cell lines C26 with 20 μM of HCF and HCT116 with 30 μM of HCF caused cell cycle arrests in the G0 phase and reduced the number of cells in the M phase. Additionally, the treated cells showed increased apoptosis rates, marked by the upregulation of the proapoptotic protein BCL-2-associated X protein (BAX) and downregulation of the anti-apoptotic protein BCL-2 [33].

TcES also directly affected human CRC cell lines alone and in combination with the chemotherapeutic agent 5-FU. In one study, exposure of the human CRC cell line RKO to 12.5 μg/mL of TcES for 72 h reduced cell numbers, although it did not affect cell viability. Furthermore, TcES induced changes in cell morphology and promoted the formation of colonospheres by reorganizing the actin cytoskeleton. Like those results observed in the AOM/DSS mouse model, TcES decreased NFκB activation in RKO cells stimulated with 0.5 mg/mL of LPS for 20 min. Notably, the inhibition of c-RAF reversed the downregulation of NFκB, suggesting that TcES may operate through the c-RAF signaling pathway [38]. Interestingly, similar effects of TcES on NFκB and c-RAF were previously observed in mouse DCs [36], and seems a possible common pathway activated by other helminth glycoconjugates, as documented in *S. mansoni* [80], and more recently in *E. granulosus* products [81], in all cases, inhibiting DC-mediated inflammatory responses.

The combined therapy using 50 μg of TcES and 10 μM of 5-FU reduced the proliferation and migration of the HCT116 and RKO cell lines. The treatment upregulated P53 and P21 proteins and decreased Y-box binding protein 1 (YB-1), a proto-oncogenic protein [76]. These findings suggest that TcES may activate tumor suppressor proteins and inhibit the activation of proto-oncogenic proteins. However, further research is necessary to understand the antitumor mechanisms of TcES.

In summary, the findings reviewed suggest that several factors influence the antitumoral effects of helminth-derived products and could involve a range of mechanisms (Figure 3). This may be due to the high complexity of their composition (several molecular compounds have been identified, including fatty acids, amino alcohols, indoles, sterols, glycosides, and sphingolipids). For this reason, it is essential to characterize and identify the components of helminth-derived products to facilitate the study of their interactions with CRC cells. Understanding these interactions may enable the identification of potential biotherapeutic molecules from helminths that improve CRC outcomes. Currently, several works describe the composition of some helminth-derived products or total antigens, including those of *E. granulosus* [82], *T. spiralis* [83], *A. lumbricoides* [37], *T. crassiceps* [84,85], among others. However, only two studies have focused on examining the effects of isolated/purified molecules from helminth-derived products on the development of CRC. These reports will be discussed in the following section.

## 5. The Impact of Isolated Molecules from Helminths in Colorectal Cancer Modulation

### 5.1. Pro-Tumorigenic Effects

Some researchers have focused on isolating specific molecules that preserve the primary biological function of whole helminth-derived products to better understand and identify them, and to reduce exposure to inactive molecules from helminths.

The molecule SjE16.7 has been identified as an EF-hand calcium-binding protein released from the eggs of *S. japonicum* and synthesized using yeast and *Escherichia coli* recombination systems. In the AOM/DSS CRC model, mice treated with 100 µg of SjE16.7 twice a week throughout the experiment showed no significant changes in body weight or colon length. However, by the 14th week after the experiment began, the mice treated with SjE16.7 exhibited a tumor incidence rate of 90 to 100%, compared to only 50 to 70% in the control cancer groups. Additionally, the colon tumors in SjE16.7-treated mice were larger than those in control mice [86].

This effect correlated with the accumulation of myeloid cells (CD11b+), Tregs, and a reduction in CD4+ and CD8+ T cells. The myeloid subset was primarily composed of Gr1^hi^ cells, a well-established marker of neutrophils [86]. These findings indicate that SjE16.7 promotes an inflammatory response associated with the progression of CRC. The immunomodulatory effects of SjE16.7 were investigated in bone marrow-derived macrophages and the RAW 264.7 macrophage cell line. Treatment with 1 µM of SjE16.7 led to increased production of ROS, activation of NFκB, and secretion of the cytokines IL-6 and TNF-α [86]. SjE16.7 is recognized by the mouse macrophage cell line RAW 264.7 and the human CRC cell lines Caco-2 and SW480 through the receptors for advanced glycation end products (RAGE). Knocking down RAGE in RAW 264.7 macrophages reduced ROS production, NFκB activation, and cytokine secretion induced by SjE16.7 [86].

As previously mentioned, ROS contributes to tissue damage and the development of colorectal tumors. In this regard, the immunomodulatory effects observed in macrophages could be proposed as a potential protumor mechanism of SjE16.7. Conversely, the increased FOXP3+ Tregs and the decreased CD4+ and CD8+ T cells indicate an immunosuppressive microenvironment mediated by SjE16.7, which may facilitate tumor progression. Given the contrasting immunomodulatory effects of SjE16.7, it is essential to investigate the patterns of cell infiltration in the colon induced by this molecule. Additionally, the recognition of SjE16.7 by CRC cell lines suggests that SjE16.7 may have a direct protumor effect.

### 5.2. Anti-Tumorigenic Effects

Another helminth-identified molecule, but with contrasting effects, is the recombinant nematode anticoagulant protein c2 (rNAPc2), which is a protein isolated initially from *Ancylostoma caninum* and expressed in the yeast *Pichia pastoris* [31]. rNAPc2 specifically inhibits the tissue factor (TF)/factor VIIa complex, which is associated with angiogenesis and metastasis [87].

The antitumor effects of rNAPc2 have been proven in several CRC mouse models. In the *APC*^min/+^ model, mice treated daily with 100 µg/kg of rNAPc2 until they reached 16 weeks of age showed reduced numbers of tumors in both the small intestine and colon compared to control mice. Additionally, histological analysis indicated a decrease in total tumor area and a lower percentage of adenocarcinomas in rNAPc2-treated mice [88].

The combination of rNAPc2 with chemotherapeutic drugs or specific antibodies has been shown to inhibit tumor formation. In a model involving human HCT116 xenograft transplantation into nude mice, those treated with rNAPc2, 5-FU, or bevacizumab (an anti-VEGF monoclonal antibody) exhibited tumor volume reduction compared to untreated mice. However, mice receiving the combined therapy rNAPc2 and 5-FU or rNAPc2 and bevacizumab demonstrated a remarkable tumor volume reduction, even greater than that observed in the individual treatments. Additionally, mice treated with these combined therapies showed reduced angiogenesis and inhibition of tumor cell proliferation [88].

The anti-metastatic effects of rNAPc2 have been demonstrated in several CRC mouse models. In a study where pulmonary metastasis was induced by injecting CT26 cells into the tail vein, mice treated with increasing doses of rNAPc2, ranging from 10 to 1000 µg/kg, exhibited reduced lung weight and a lower number of surface metastases compared to untreated controls. Additionally, in a liver metastasis model where human HCT116 cells were administered via the portal vein to nude mice, those treated with rNAPc2 or the chemotherapeutic irinotecan (CPT-11) showed a decrease in tumor areas in the liver. Notably, the anti-metastatic effect was enhanced when rNAPc2 and CPT-11 were given together [88].

As mentioned above, rNAPc2 inhibits the TF complex, which is associated with angiogenesis and metastasis. For these reasons, authors have proposed that rNAPc2 antitumor effects may be linked to the surface expression of TF on tumor cells. Experiments using HCT116 cells with TF knockdown showed that these cells have slower growth in nude mice xenografts compared to HCT116 parental cells. Interestingly, tumors induced by HCT116 TF knockdown cells are resistant to the antitumor effects of rNAPc2 compared to HCT116 parental cells.

**Figure 4:**
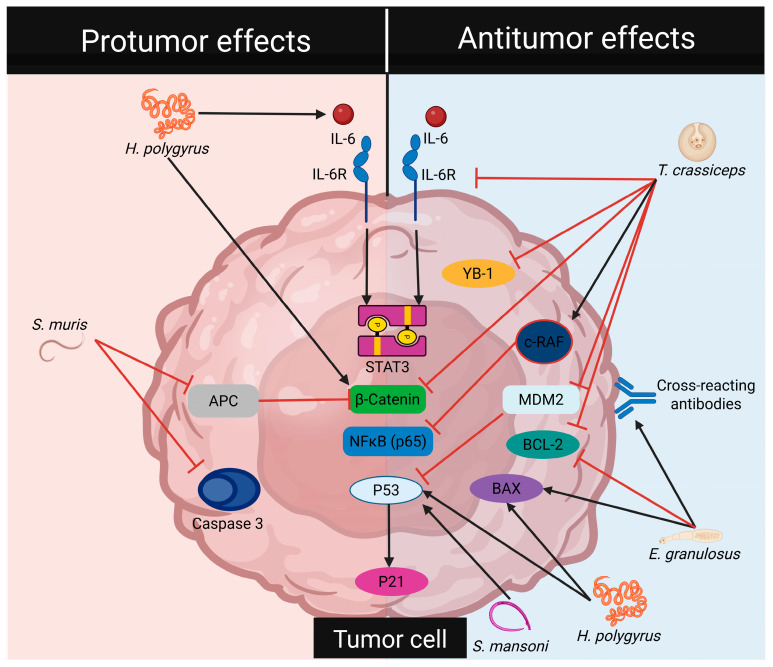
Helminths regulate cell signaling pathways in tumor cells. Pro-tumorigenic effects of helminths involve the activation of transcription factors associated with cell proliferation and the inhibition of pro-apoptotic proteins. In contrast, anti-tumorigenic effects of helminths inhibit the activation of transcription factors related to cell proliferation, downregulate anti-apoptotic proteins, and upregulate pro-apoptotic proteins.

In summary, the reviewed literature indicates that helminths can have both negative and positive effects on the initiation and progression of CRC (Table 1, Figure 2 and Figure 3). Currently, research has mainly focused on harnessing the antitumor properties of helminths. However, studies involving helminth-derived products as a potential treatment for CRC are still limited to basic science research and preclinical trials, predominantly conducted using animal models and in in vitro cultures of human colorectal tumor cells. While these models provide valuable insights, they capture only a small fraction of the complex mechanisms involved in CRC development. Nevertheless, advancements in technology have led to the development of new models, such as genetically engineered mouse models, which enable more comprehensive research [41]. It is crucial to underscore the mechanisms of action shared by different helminths and their products on CRC modulation. These mechanisms, include the downregulation of inflammatory responses (consistent inhibition of TNFα and IL-17), blockade of STAT3 phosphorylation (activation of phosphatases?), and induction of proapoptotic proteins, along with reactivation of P53 that modulates cell proliferation, as depicted in Figure 4, appear to be genetic background independent, as at least BALB/c and C57BL/6 mice can benefit from these treatments.

On the other hand, ongoing clinical trials are investigating the use of helminth-derived products for the treatment of several inflammatory and autoimmune diseases, such as multiple sclerosis and IBD. In these trials, the efficacy and safety of helminth-derived products have been demonstrated, with some yielding successful results [89]. For instance, a pilot Phase 2a clinical trial evaluated the protein P28 glutathione-S-transferase, which resulted in a reduced Crohn’s Disease Activity Index without severe adverse events, limited to local reactions at the injection site and mild gastrointestinal disorders [90].

Thus, it is not impossible to achieve this in a few years, once isolated or enriched helminth-derived products are molecularly better characterized and tested in CRC.

## 6. Concluding Remarks

In this review, we gathered evidence on how helminths influence outcomes in CRC. Helminths and their products can either promote or inhibit colonic tumor progression depending on various factors, such as the species of helminths, their primary site of localization, the origin of the colorectal tumor, and the stage of the disease. While several physiological changes have been observed in mice bearing CRC when infected with helminths or treated with helminth-derived products, the exact mechanisms impacting CRC outcomes are not yet fully understood. One of the most studied mechanisms is immune modulation. Evidence suggests that in inflammation-associated cancer models, the balance between immunosuppression and exacerbation of inflammation mediated by helminths is a key factor in tumor progression during the early stages of the disease. Specifically, helminths that exacerbate inflammation may promote tumor formation, while those that suppress immune responses may inhibit it. Conversely, in hereditary CRC models, immunosuppression induced by helminths could promote tumorigenesis. This highlights the importance of considering both the origin and stage of the tumor in evaluating the effects of helminths on CRC.

Given that helminth therapy using whole parasites is impractical and could compromise patient health, recent research has focused on helminth-derived products as a more viable therapeutic approach. The extraction of helminth-derived products has enabled the study of new mechanisms by which these organisms may impact CRC.

Like active helminth infections, the investigation of ES helminth products as immune modulators has been extensively studied. Antitumor effects of these products have been linked to both the stimulation and the suppression of immune responses. Evidence indicates that the immune stimulatory effects of helminth-derived products may occur through cross-reactivity between helminth and cancer antigens. Conversely, ES helminth products may downregulate key inflammatory transcription factors that favor CRC.

Recent studies have also demonstrated that, in addition to immunomodulatory mechanisms, helminth-derived products could interact directly with CRC cells. In this context, ES helminth products have shown the potential to either promote or inhibit cell proliferation and cell death. Tumor-promoting effects are associated with the activation of proto-oncogenes and the inhibition of anti-apoptotic proteins, while antitumor effects are linked to the upregulation of tumor suppressor proteins (Figure 4).

One of the main challenges in utilizing ES helminth products as potential biotherapeutics lies in the complexity of their composition. As a complex mixture of biomolecules, studying their interaction with tumor cells can be difficult. Further characterization and identification of the components of ES helminth products will allow researchers to investigate their interaction with specific receptors in immune, tumor, and epithelial cells, aiding in the development of potential biotherapeutic agents. Nevertheless, we must consider potential adverse effects, such as immunosuppression or the induction of PDL-1 and PD-L2 expression, that may compromise the use of immunotherapy aimed at blocking immunologic checkpoints.

Research on helminth-derived products as a potential treatment for CRC is currently limited to basic science studies and preclinical trials conducted in animal models and in vitro cultures using human colorectal tumor cells. While these models provide valuable insights, they only capture a small fraction of the complex mechanisms involved in the development of CRC. It is important to note that accurately reproducing the development and progression of CRC is challenging due to the multifactorial nature of the disease. CRC is a heterogeneous condition, meaning there are numerous differences among patients’ tumors, and even variations in tumors from the same patient. Thus, there is no ideal in vitro or preclinical model for any human disease. However, numerous efforts are underway to develop preclinical models that closely resemble human diseases. Several models attempt to replicate human disease in colorectal cancer as closely as possible. The models described in this review are limited and involve chemical inducers, injecting syngeneic heterotopic tumor cells, single-gene mutations, and the induction of inflammation. They all have limitations in several aspects, such as the development of a limited number of mutations, restricted tumor heterogeneity, and poor metastasis development. However, these preclinical models have generated valuable information for understanding the development of CRC in humans, particularly in terms of the role of innate and adaptive immune response, chronic inflammation, and insights into the tumor microenvironment. There are more preclinical models of CRC. However, with the advent of genetically modified animals, it has become possible to develop models with up to four CRC-related mutations in the same animal, thereby generating a model that more closely resembles the classification of the four consensus molecular subtypes described in human CRC. Furthermore, organoids or tumor cells from patients have been grafted into immunodeficient mice to investigate more effective treatments for the patients, thereby providing precision medicine, which aims to develop therapeutic strategies tailored to the specific characteristics of tumors. However, these aforementioned models have not yet been addressed from the perspective of treatment with helminth-derived molecules. Thus, this is a point that researchers in this field should develop soon to solidify the potential beneficial effects of using helminth ES products. Finally, as mentioned before, the use of helminth infection is not an option, given the potential harmful for the patients; we propose instead the use of helminth-derived products, not to displace the conventional drugs in CRC (even when their efficacy is low) but the combination of such products with standard drugs to improve their efficacy and reduce the drug doses to decrease the severe side effects in patients. Understanding how these products impact CRC is vital for advancing translational research that can identify new therapeutic targets.

## Figures and Tables

**Table 1:** Effects of helminths and their products on different models of CRC.

Pro-Tumorigenic Effects				
Infection				
Helminth	Model	Clinical Findings	Molecular Findings	Ref
*Heligmosomoides polygyrus*	Mouse AOM/DSS	Accelerated weight loss and higher incidence of tumors.	Upregulation of IL-6 and CXCL1.	[20]
*Schistosoma japonicum*	CRC patients	Egg depositions around CRC tumors.	ND	[21,22]
	CRC patients	ND	Upregulation of VEGF and PD-ECGF in CRC tumors.	[23]
*Schistosoma mansoni*	Mouse infection	Distortion of colonic crypts, glandular and mucosal dysplasia, nuclear hyperchromasia, and serration of the surface epithelium.	Upregulation of PTX3, TNF-α, and iNOS.	[24]
*Schistosoma sp*	CRC patients	Reduction of overall survival rates.	ND	[25,26]
*Syphacia muris*	Rat DMH	Increased epithelial cell proliferation and aberrant crypt formation.	Downregulation of caspase 3 and APC. Upregulation of COX2.	[27]
*Trichuris muris*	Mouse infection	Colon hyperplasia, development of aberrant crypts and pre-adenomas, influx of inflammatory cells into the colonic lamina propria.	Upregulation of IL-6, TNF-α, and IFN-γ.	[28]
	Mouse AOM	Increased levels of neoplasia scores.	ND	[28]
	Mouse *APC*^min/+^	Accelerated tumor formation in the large intestine.	ND	[28]
**Helminth-Derived Products**				
**Helminth**	**Model**	**Clinical Findings**	**Molecular Findings**	**Ref**
*Cooperia curticei* (Conditioned medium)	In vitro HT29-D4 (human)	Increased cell proliferation.	ND	[29]
*Heligmosomoides polygyrus* (HES)	In vitro CT26 (mouse)	Increased cell migration.	Increased expression of β-catenin.	[29]
*Trichostrongylus colubriformis* (Conditioned medium)	In vitro HT29-D4 (human)	Increased cell proliferation.	ND	[30]
*Trichostrongylus vitrinus* (Conditioned medium)	In vitro HT29-D4 (human)	Increased cell proliferation.	ND	[29]
**Isolated Molecules**				
**Helminth**	**Model**	**Clinical Findings**	**Molecular Findings**	**Ref**
SjE16.7 (*Schistosoma japonicum*)	AOM/DSS	Increased tumor incidence rate and tumor size.	Increased production of ROS, upregulation of NFκB, IL-6, and TNF-α. Increased FOXP3+ Tregs and decreased CD4+ and CD8+ T cells.	[31]
**Anti-Tumorigenic Effects**				
**Infection**				
**Helminth**	**Model**	**Clinical Findings**	**Molecular Findings**	**Ref**
*Taenia crassiceps*	AOM/DSS	Reduction in size and number of tumors, low-grade dysplasia, and preservation of goblet cells.	Recruitment of AAMs, downregulation of CXCR2, iNOS, and TNF-α. Upregulation of IL-4.	[32]
**Helminth-Derived Products**				
**Helminth**	**Model**	**Clinical Findings**	**Molecular Findings**	**Ref**
*Ascaris lumbricoides* (ALES)	In vitro HCT116 (human)	Inhibition of cell proliferation.	ND	[33]
*Echinococcus granulosus* (HCF)	Syngeneic heterotopic CT26 (mouse)	Reduction in the mean tumor area.	Downregulation of IL-2, TNF-α, and IFN-γ. High levels of IgG.	[34]
	Syngeneic heterotopic CT26 (mouse)	Reduction in tumor incidence. Increased mouse survival.	High production of anti-HCF IgG that recognizes HSP70.	[35]
	In vitro C26 (mouse)	Cell cycle arrests.	Upregulation of BAX. Downregulation of BCL-2.	[36]
	In vitro HCT116 (human)	Cell cycle arrests.	Upregulation of BAX. Downregulation of BCL-2.	[36]
*Heligmosomoides polygyrus* (HES)	In vitro CT26 (mouse)	Decreased viable cell counts, reduced DNA synthesis, and reduced mitochondrial activity.	Upregulation of P21 and P53.	[37]
	In vitro HCT116 (human)	Reduced cell viability and DNA synthesis.	Upregulation of P21 and P53.	[37]
*Nematodirus battus* (derived products)	In vitro HT29-D4 (human)	Inhibition of cell proliferation.	ND	[29]
*Schistosoma mansoni* (ASMA)	Mouse DMH	Increased overall survival rates. Reduction in the tumor size, the number of neoplastic lesions, and the average lesion size.	Low levels of IL-17.	[38]
*Taenia crassiceps* (TcES)	AOM/DSS	Inhibition of colonic tumor formation in 45% of mice. Reduction in tumor size. Normal colon epithelium morphology and maintained normal goblet cell counts.	Downregulation of STAT3, DNMT1, Cyclin D, β-catenin, NFκB, and BCL-2. Reduced levels of IL-1β, IL-17, IL-23, IL-33, CXCR2, and ICAM-1.	[39]
	AOM/DSS	Synergistic effect with 5-FU. Reduced colonic tumor load.	Increased infiltration of NK cells. Reduced expression of MDM2. Upregulation of P53 and P21.	[40]
	In vitro RKO (human)	Reduced cell proliferation. Formation of colonospheres by reorganizing the actin cytoskeleton.	NFκB downregulation via c-RAF activation.	[39]
	In vitro RKO and HCT116 (human)	Synergistic effect with 5-FU.	Upregulation of P21 and P53. Decreased expression of YB-1.	[40]
*Trichinella spiralis* (ATSA)	Mouse DMH	Increased overall survival rates.	Low levels of IL-17	[38]
**Isolated Molecules**				
**Helminth**	**Model**	**Clinical Findings**	**Molecular Findings**	**Ref**
rNAPc2 (Initially isolated from *Ancylostoma caninum*)	Mouse *APC*^min/+^	Reduced number of tumors. Decreased total tumor area and a lower percentage of adenocarcinomas.	ND	[41]
	Xenograft HCT116 (human)	Synergistic effect with 5-FU and bevacizumab. Tumor volume reduction, reduced angiogenesis, and inhibition of tumor cell proliferation.	ND	[41]
	Allograft lung metastasis CT26 (mouse)	Reduced lung weight and a lower number of surface metastases.	ND	[41]
	Xenograft liver metastasis HCT116 (human)	Synergistic effect with CPT-11. Decreased surface metastases in the liver.	ND	[41]

## Data Availability

Not applicable.

## Abbreviations

The following abbreviations are used in this manuscript:

5-FU	5-Fluorouracil
AAMs	Alternatively activated macrophages
ALES	Ascaris lumbricoides ES products
AOM	Azoxymethane
APC	Adenomatous polyposis coli
ASMA	Autoclaved Schistosoma mansoni antigens
ATSA	Autoclaved Trichinella spiralis antigens
BAX	BCL-2-associated X protein
BCG	Bacillus Calmette-Guerin
BCL-2	B-cell lymphoma 2
CCR2	C-C chemokine receptor 2
CMS	Consensus molecular subtypes
COX2	Cyclooxygenase 2
CPT-11	Irinotecan
CRC	Colorectal cancer
CXCL1	Chemokine (C-X-C motif) ligand 1
CXCR2	C-X-C chemokine receptor 2
DCs	Dendritic cells
DMH	1,2-dimethylhydrazine
DNMT1	DNA methyltransferase 1
DSS	Dextran sulfate sodium
ES	Excreted/secreted
FAP	Familial adenomatous polyposis syndrome
HCF	Hydatid cyst fluid
HES	Heligmosomoides polygyrus excreted/secreted products
HSP70	Heat shock protein 70
IARC	International Agency for Research on Cancer
IBD	Inflammatory bowel diseases
ICAM-1	Intercellular adhesion molecule 1
IECs	Intestinal epithelial cells
IFN-γ	Interferon gamma
Ig E/G/G1/G2a	Immunoglobulin E/G/G1/G2a
IL-1β/2/4/5/6/9/10/13/17/17F/23/25/33	Interleukin 1β/2/4/5/6/9/10/13/17/17F/23/25/33
IL-6R	IL-6 receptor
ILC2	Type 2 innate lymphocytes
iNOS	Inducible nitric oxide synthase
MDM2	Murine double minute 2
MYC	Myelocytomatosis
NFκB	Nuclear factor kappa-light-chain-enhancer of activated B cells
NK	Natural killer cells
PDECGF	Platelet-derived endothelial growth factor
PTX3	Pentraxin 3
RAGE	Receptors for advanced glycation end products
rNAPc2	Recombinant nematode anticoagulant protein c2
ROS	Reactive oxygen species
STAT3	Signal transducer and activator of transcription 3
TcES	Taenia crassiceps excreted/secreted products
TF	Tissue factor
TGF-β	Transforming growth factor beta
Th 1/2/17	T helper 1/2/17
TNF-α	Tumor necrosis factor-alpha
Tregs	T regulatory cells
TSLP	Thymic stromal lymphopoietin
UC	Ulcerative colitis
VEGF	Vascular endothelial growth factor
WNT	Wingless-related integration site
YB-1	Y-box binding protein 1
